# Comparative Experimental Evaluation of Orthodontic Appliances for Maxillary Arch Expansion

**DOI:** 10.3390/jcm13216473

**Published:** 2024-10-29

**Authors:** Ghazal Ebrahimy, Anna Konermann, Tarek El-Bialy, Ludger Keilig, Christoph Bourauel

**Affiliations:** 1 Oral Technology, University Hospital Bonn, 53111 Bonn, Germany; 2 Department of Orthodontics, University Hospital Bonn, 53111 Bonn, Germany; 3 Division of Orthodontics, School of Dentistry, Faculty of Medicine and Dentistry, University of Alberta, Edmonton, AB T6G 2R3, Canada; 4 Department of Prosthodontics, University Hospital Bonn, 53111 Bonn, Germany

**Keywords:** arch expansion, orthodontic forces, posterior crossbite, quadhelix, torque

## Abstract

**Background/Objectives:** The orthodontic treatment of posterior crossbite using appliances for gradual maxillary expansion is crucial to ensure proper transversal jaw relationships as much as occlusal functionality. The aim of this study was to analyze forces and torques generated by different appliances for maxillary expansion. **Methods:** Measurements were conducted for the Wilson^®^ 3D^®^ Quadhelix (WQH) and Wilson^®^ 3D^®^ Multi-Action Palatal Appliance (WPA) across various sizes and compared to the Remanium^®^ Quadhelix (RQH). Activations were set to 8 mm for the WQH and RQH and 6 and 8 mm for the WPA. Rotations and root torque were simulated via an activation of 10° for arches. A total of eight test series were conducted. **Results:** The WPA displayed the highest force and torque values for all movements, far surpassing recommended guideline values (expansion 8.5–>15.0 N/46.3–86.5 Nmm, rotation 3.1–6.1 N/40.7–61.4 Nmm, torque 3.9–5.1 N/22.4–29.7 Nmm), and the WQH displayed the lowest values (expansion 2.7–12.6 N/11.1–39.6 Nmm, rotation 0.1–1.7 N/23.0–32.2 Nmm, torque 0.9–2.9 N/3.4–10.5 Nmm). Appliances with the smallest transverse dimensions exhibited the highest force and torque maxima. **Conclusions:** This study underscores the importance of understanding biomechanical principles in orthodontics for minimizing unintended tooth movements, providing detailed insights into the force systems of appliances acting in the transverse plane, and establishing a foundation for future clinical investigations to validate these in vitro findings.

## 1. Introduction

Posterior crossbite is a prevalent malocclusion in primary and mixed dentition, affecting approximately 5% to 8% of children aged 3 to 12 years [1]. This orthodontic dysgnathia arises from skeletal malocclusion, dentoalveolar malocclusion, or a combination of both, manifesting as a unilateral or bilateral crossbite of individual or groups of posterior teeth (dental crossbite), malocclusion of posterior teeth coupled with a functional displacement of the mandible (functional crossbite), or a transverse discrepancy between the maxilla and mandible (skeletal crossbite) [2,3]. Orthodontic intervention for correcting a crossbite is crucial to prevent adaptive processes in the temporomandibular joint, as studies have demonstrated a correlation between malocclusion and temporomandibular joint disorders [4,5]. Furthermore, untreated crossbites can result in restricted maxillary growth and subsequent functional disorders due to skeletal manifestation. The objective of early treatment is to restore ideal dental and skeletal relationships, enhancing chewing function and ensuring a symmetrical alignment between the condyle and fossa.

The therapeutic approaches for the treatment of posterior crossbites are diverse and contingent upon the specific malocclusion type, its severity, concurrent orthodontic issues, and the timing of treatment. Investigations revealed a preference for the technique of slow maxillary expansion (SME) over rapid maxillary expansion (RME) in the treatment of posterior crossbites if applicable and within the scope of treatment, as SME applies relatively small and gradually increasing forces over an extended period, facilitating a more physiological adaptation of the supporting tissues and theoretically reducing the risk of relapse [6]. Even if only dental expansion is achieved, sufficient growth can induce slight skeletal changes as well [6]. The benefits of SME include the application of a consistent physiological force, the prevention of anterior tooth tipping, and minimal stress on the anchored teeth. SME has been shown to enhance post-expansion stability, provided that an adequate retention phase is maintained [7]. In growing patients, SME has been shown to significantly reduce pain levels during the initial week of treatment when compared to RME, whereas the latter one is considered one of the most painful early orthodontic procedures, as evidenced by the intensity of symptoms reported by patients [8,9]. Compared to rapid activation, slow activation demonstrates a substantial improvement in the overall patient experience [10]. However, its primary drawback is the longer treatment duration, extending over several months compared to RME [6]. Moreover, another advantage of RME is its resultant minimal dental movement, as the rapid, high-magnitude forces applied to the posterior teeth provide insufficient time for significant dental movement, leading to force transmission to the cranial sutures. Once the applied force surpasses the threshold necessary for orthodontic tooth movement and sutural resistance, the sutures begin to separate, while the teeth exhibit only minimal displacement relative to the alveolar bone [7]. RME is recommended for patients with a transverse skeletal discrepancy of at least 4 mm, particularly when the maxillary molars demonstrate buccal angulation as a compensatory mechanism for the underlying maxillary constriction [7].

The quadhelix appliance, designed for the treatment of a posterior crossbite effected by reduced maxillary width, is a highly effective device for correcting malocclusion [11,12]. Its success is further ensured by its fixed nature, which does not rely on patient compliance. It is primarily utilized for modest expansions of the maxilla and can furthermore affect distalization, molar rotation, and torque adjustments [7,13]. This method of SME also encourages a more natural response within the median palatal suture area, minimizing tissue resistance due to consistent force distribution and promoting improved ossification [9].

Several designs of quadhelix appliances exist to date, each with specific modifications aimed at optimizing treatment outcomes. The aim of this study was to analyze potential differences in the force and torque values generated by various quadhelix designs, assessing their efficacy in correcting posterior crossbites and facilitating maxillary expansion.

The central hypothesis of this investigation posited that all appliances evaluated for transversal maxillary expansion would exhibit equivalent biomechanical properties, specifically in terms of the force and torque generated during expansion, rotation, and root torque application. It was anticipated that, despite potential variations in design and structural characteristics, the appliances would demonstrate equivalent mechanical behavior when subjected to standardized testing conditions. This hypothesis aimed to assess whether the devices could achieve similar therapeutic outcomes, thereby ensuring uniformity in clinical efficacy for maxillary expansion across different appliances.

## 2. Materials and Methods

### 2.1. Appliances

**Figure 1:**
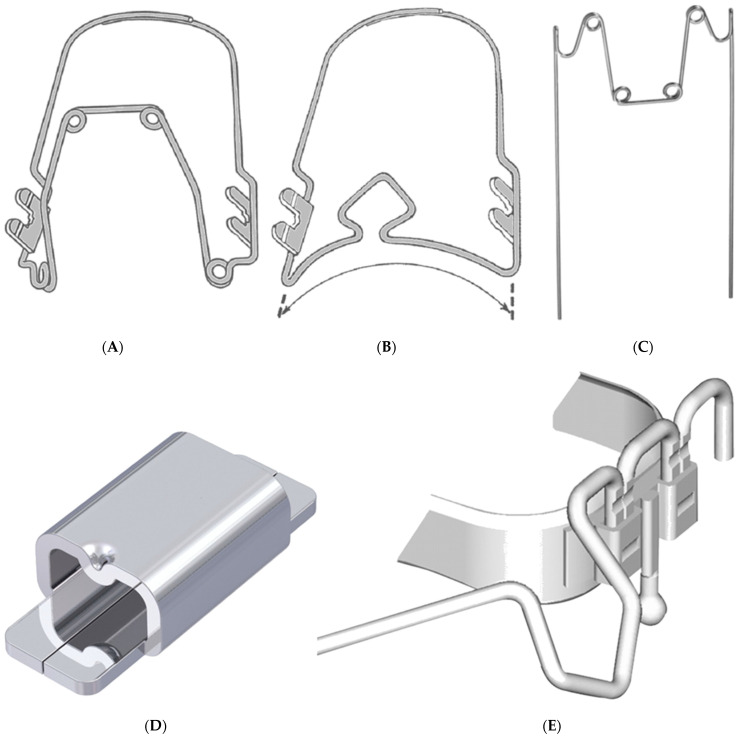
The three appliances investigated for the resulting forces and torques induced by experimental expansion, rotation, and root torque. Wilson^®^ 3D^®^ Quadhelix (left, (**A**)), Wilson^®^ 3D^®^ Multi-Action Palatal Appliance (middle, (**B**)), Remanium^®^ Quadhelix (right, (**C**)). A Rocky Mountain Orthodontics^®^ (RMO) lock, pre-welded for vertical insertion, used for the molar bands of the Wilson^®^ 3D^®^ appliances (left, (**D**)). An Orthorama^®^ (Dentaurum) lock bonded directly to the Frasaco teeth for the horizontal insertion of the Remanium^®^ Quadhelix (right, (**E**)).

The forces and torques induced by experimental expansion, rotation, and root torque were systematically analyzed for the Wilson^®^ 3D^®^ Quadhelix (WQH) and the Wilson^®^ 3D^®^ Multi-Action Palatal Appliance (WPA) (Rocky Mountain Orthodontics^®^, Franklin, TN, USA) and compared with the Remanium^®^ Quadhelix (Dentaurum, Ispringen, Germany) (RQH). The three different appliances were investigated in various sizes, each listed in Table 1. The Wilson^®^ 3D^®^ appliances represent an advanced product line available in a wide range of sizes, incorporating a 0.9 mm round wire and a novel “plug-in/plug-out” connector system that allows for insertion into molar locks without the need for soldering or welding. The WQH is manufactured from Blue Elgiloy^®^, whereas the WPA is made from stainless steel with a chromium nickel base. The Wilson^®^ 3D^®^ appliances are equipped with welded twin pins to ensure secure attachment within the brackets. The three appliances examined in this study are illustrated in Figure 1A–C.

### 2.2. The Preparation of the Test Specimens

To determine the forces and torques, two Frasaco plastic molars were fitted with molar bands for each appliance. Rocky Mountain Orthodontics^®^ (RMO) locks (RMO, Franklin, IN, USA), pre-welded for vertical insertion, were used for the Wilson^®^ 3D^®^ appliances (Figure 1D). In contrast, for the RQH, Orthorama^®^ (Dentaurum) locks were bonded directly to the teeth without molar bands to allow for the horizontal insertion of the appliance (Figure 1E). Subsequently, the teeth were attached to the force/torque sensors of the Orthodontic Measurement and Simulation System (OMSS) as previously described [14]. In brief, the system comprises two force/torque sensors capable of measuring forces and torques in all three spatial planes simultaneously.

### 2.3. Experimental Procedure

Tooth movements involving expansion, rotation, and root torque were simulated in three distinct test series. Five specimens per appliance variant and size were examined, with four individual measurements conducted for each to ensure the detection of potential errors through repetition. Table 2 outlines the appliances investigated, including their respective activation parameters and the corresponding simulations of tooth movement. As the first test series, the expansion movement of the appliances was performed with the activation set to 8 mm for the quadhelical appliances and between 6 and 8 mm for the palatal appliances. The second series of tests involved simulating rotational tooth movement with an activation of 10° for the arches. In the final series, root torque was simulated, with the two Wilson^®^ 3D^®^ appliances activated at 10°. Due to the round wire design of the RQH, root movement simulation was not possible for this appliance.

### 2.4. Data Analysis and Statistics

The measured values of the force and torque maxima determined with the OMSS were evaluated using Excel Version 2016. Despite fine adjustments that needed to be made to achieve a zero-force system prior to testing, minor forces and torques with maximum values of 0.2 N and 15 Nmm, respectively, were present before the test. Consequently, an offset correction was applied during the evaluation to ensure that the initial forces and torques had a value of zero. The subsequent values obtained from the five samples per appliance variant, along with their mean values and standard deviations, were tested for normal distribution using the Kolmogorov–Smirnov test. Following this, significant differences were assessed using Student’s *t*-tests with a significant level of α = 0.05. *p* < 0.05 was considered statistically significant. To account for potential alpha error accumulation, a Bonferroni correction was applied post hoc, and the corrected significance level was α = 0.00048.

## 3. Results

Investigations revealed that the measured forces for the different appliances often surpassed the guideline values recommended in the literature, with the WPA exhibiting the highest and the WQH the lowest force values. Table 3 provides a direct comparison of the measured force values against the guideline values reported in the literature.

The measurement results of the force and torque values for each type and dimension of the three appliances encompassing the different activation conditions showed significant differences, which are presented in comprehensive detail below. Consequently, the null hypothesis positing that all examined appliances should exhibit equivalent performance was rejected.

### 3.1. Expansion

**Figure 2:**
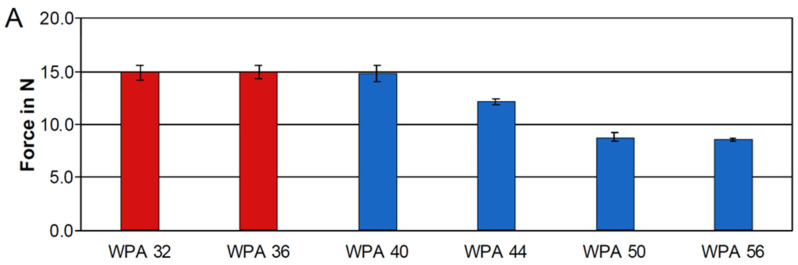

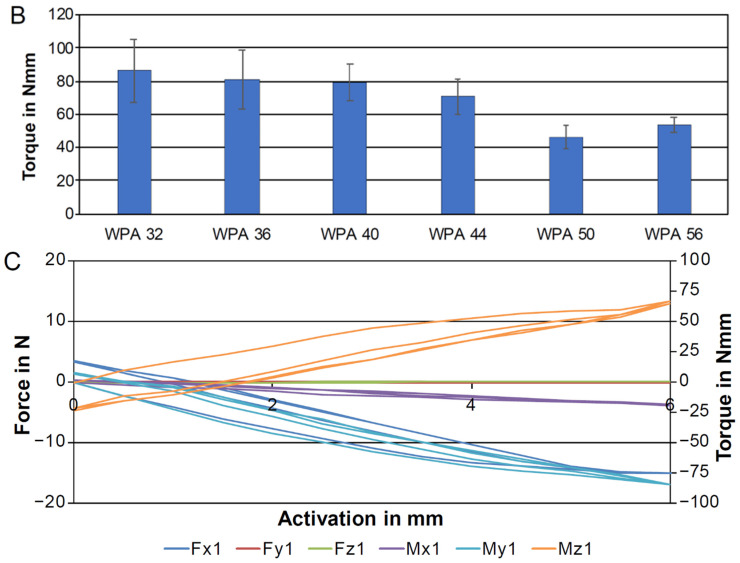
The forces measured in Newtons (N) (**A**) and torques measured in Newton-millimeters (Nmm) (**B**) exerted by the Wilson^®^ 3D^®^ Multi-Action Palatal Appliance (WPA) during expansion activation recorded by the OMSS are presented in columns corresponding to each appliance size, ranging from 32 mm to 56 mm. Forces exceeding 15 N, which surpassed the measurement capacity of the OMSS’s sensors, are highlighted in red. Exemplified force and torque components during an expansion movement with a WPA size of 32 mm (**C**). A total of *n* = 5 specimens per appliance variant and size were analyzed.

The analyses of the expansion movements revealed that the force maxima for the WPA extremely surpassed the recommended value from the literature of 1.2 N for all dimensions investigated. An activation of 6 mm was performed only with the first variant of the WPA of size 32 mm, as an activation of 8 mm already resulted in forces exceeding 15 N, which could not be recorded by the OMSS’s sensors. During the activation measurement, forces ranged from 8.5 t to more than 15.0 N with standard deviations between 0.1 and 0.7 N, while torques ranged from 46.3 to 86.5 Nmm with standard deviations between 4.5 and 18.9 Nmm. The results are graphically illustrated in Figure 2A,B. The force and torque components during an expansion movement with a WPA size of 32 mm are exemplified in the graph shown in Figure 2C.

**Figure 3:**
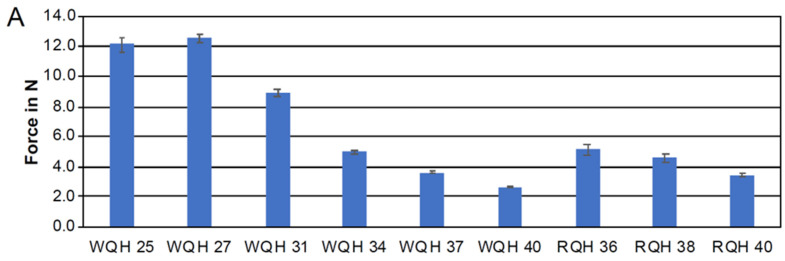

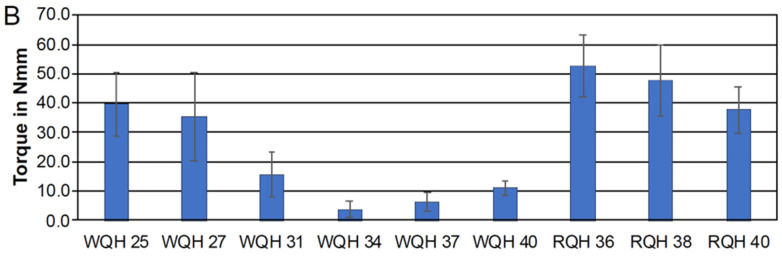
Forces measured in Newtons (N) (**A**) and torques measured in Newton-millimeters (Nmm) (**B**) exerted by the Wilson^®^ 3D^®^ Quadhelix (WQH) across sizes ranging from 25 to 40 mm and for the Remanium^®^ Quadhelix (RQH) across sizes ranging from 36 to 40 mm. A total of *n* = 5 specimens per appliance variant and size were analyzed.

The mean peak forces of the WQH at an 8 mm activation ranged from 2.7 to 12.6 N, with standard deviations between 0.1 and 0.5 N, as presented in Figure 3A. While these values exceeded the levels recommended in the literature as well, they were still significantly lower compared to those of the other devices studied. Correspondingly, the resulting torques varied from 11.1 to 39.6 Nmm, with standard deviations between 0.1 and 0.5 Nmm (Figure 3B). In contrast, the RQH exhibited forces ranging from 3.5 to 5.2 N, with standard deviations between 0.1 and 0.4 N, and torques ranging from 37.6 to 52.6 Nmm, with standard deviations between 0.1 and 0.4 Nmm. The results moreover indicated that the appliance variants with the smallest transverse dimensions exhibited the highest force and torque maxima. A detailed comparison of the force maxima for the quadhelices showed a notable difference between the WQH and the RQH, with the small sizes of the WQH exhibiting extremely high forces but lower values compared to the RQH when looking at the appliance variant of size 40 mm with 2.7 N.

### 3.2. Rotation

The pattern of rotation values obtained for the devices was analogous to that of the expansion values. For the WPA, again, the highest force measurements could be observed, varying from 3.1 to 6.1 N with standard deviations between 0.2 and 0.4 N. The maximum torques recorded for this appliance ranged from 40.7 to 61.4 Nmm with standard deviations from 9.9 to 13.4 Nmm. The maximum rotational forces observed for the WQH ranged from 0.1 to 1.7 N, with standard deviations between 0.1 and 0.2 N. Notably, the lower end of this range was significantly below the recommended value of 0.6 N, as cited in the literature. The RQH displayed forces from 1.4 to 1.8 N with a standard deviation of 0.1 to 0.2 N. Upon analyzing the appliance sizes, the RQH with a dimension of 36 mm generated greater forces, reaching up to 1.8 N, in contrast to the WQH with a dimension of 37 mm, which produced forces measuring 0.4 N. The mean torque maxima for the RQH were between 28.2 and 32.0 Nmm with standard deviations of 7.6 to 10.1 Nmm. In comparison, the WQH exhibited again the lowest torque values ranging from 23.0 to 32.2 Nmm, with standard deviations from 3.3 to 6.9 Nmm. Overall, the analysis revealed that the WPA variants with the smallest transverse dimensions exhibited the highest force maxima. In contrast, the RQH displayed a consistent force/torque ratio across all examined sizes. For the WQH, the force and torque values decreased from the smallest to the medium-sized variant of 34 mm, then again increased with larger transverse dimensions.

### 3.3. Torque

The results of the simulated torque movement for the Wilson^®^ 3D^®^ appliances are detailed below. A comparable torque movement with the RQH was not feasible due to the inherent round wire of the system. The average force and torque maxima for the WPA during activation measurement were 3.9 to 5.1 N with standard deviations from 0.3 to 0.6 N, and the torque values ranged from 22.4 to 29.7 Nmm with standard deviations between 5.6 and 9.1 Nmm. During the 10° root torque movement of the WQH, the force maxima were distinctly lower than those for the WQH and ranged from 0.9 to 2.9 N with standard deviations between 0.1 and 0.3 N, and the mean torque maxima between 3.4 and 10.5 Nmm, with standard deviations from 1.6 to 6.1 Nmm, were markedly lower than those for the WPA. All root torque movement measurements exhibited uniform force/torque ratios that could not be correlated with the transverse dimension of the appliance. Notably, all Wilson^®^ 3D^®^ devices demonstrated high standard deviations in torque values, indicating considerable variability in their performance.

### 3.4. A Statistical Evaluation of the Force and Torque Results in an Expansion with Maxillary Appliances

The statistical analyses indicated that most of the appliances examined exhibited significant differences in force development. As visible in Table 4, only in five instances could no significant differences in expansion be observed, such as between the WQH size of 25 mm and the WPA size of 50 mm or between the WQH size of 31 mm and the WPA size of 50 mm. Notably, the evaluations demonstrated significant differences between the WPA and the RQH, as well as between the WQH and the RQH. Only one case, the WQH size of 34 mm compared to the RQH size of 36 mm, showed no significant difference.

The statistical analysis of the torque results for expansion shown in Table 5 revealed that most of the appliances differed significantly. Significant differences were observed between the WPA variants of sizes 32 mm, 36 mm, and 40 mm compared to all WQH and RQH appliances investigated. Moreover, the WQHs of sizes 31 mm, 37 mm, and 40 mm exhibited significant differences compared to the conventional RQH across all dimensions.

These findings underscore the importance of considering appliance size and variant when evaluating orthodontic force and torque characteristics.

## 4. Discussion

Orthodontic treatment is influenced by a multitude of factors, including growth dynamics and tissue responses to applied orthodontic devices, which inherently exhibit a degree of unpredictability and lack of complete control. However, the mechanical forces exerted on teeth by these devices represent a controllable variable, and this advantage should be utilized appropriately [16]. The inadvertent movements of teeth are frequently attributable, either directly or indirectly, to an insufficient comprehension of the biomechanical principles underlying orthodontic forces. This study seeks to address this gap by systematically investigating and comparing the forces and torques generated by three distinct types of transversally acting orthodontic appliances, none of which are expected to induce maxillary suture opening. This comprehensive analysis aims to elucidate the biomechanical behaviors of these devices, thereby enhancing the precision and predictability of orthodontic treatments aiming at transversal maxillary arch expansion. In the current investigation, the force systems produced by the Wilson^®^ 3D^®^ appliances and the RQH appliances were experimentally analyzed using FE simulations.

The findings from these investigations indicate that the WPA demonstrated notably higher force and torque magnitudes compared to the other appliances examined with regard to all tooth movements. Previous studies have cautioned against expanding conventional quadhelices beyond 4 to 5 mm due to the risk of inducing forces and torques that exceed physiological limits, such as a force of 2.5 N with a 5 mm transverse expansion [17]. However, the force values of the WPA cannot be solely justified by the activation distance of 8 mm, as the two other devices examined were also activated by this amount and did not exhibit comparably high values. Furthermore, the appliance variants of both the WQH and RQH with the larger transverse dimension generally achieved physiological force values, specifically measuring 3.7 N and 2.7 N for the WQH sizes of 37 mm and 40 mm, respectively. This underscores the existing literature indicating that the force transmission of quadhelices is contingent upon their dimensions, with an observed reduction in force as the appliance size increases for a consistent arch wire diameter [18]. It is well documented that excessive forces can lead to adverse effects on dental and periodontal tissues, correlating with phenomena such as root resorption and periodontal damage [19,20]. Thus, the differential force profiles exhibited by these appliances underscore the critical importance of knowledge of these parameters for carefully managing force application in orthodontic treatment.

Regarding the torque outcomes, the measurements revealed a broad spectrum ranging from low to exceedingly high values. According to the literature, therapeutic torques should ideally remain below 20 Nmm to avoid undesired effects [17]. Consistent with the findings on force magnitudes, the appliances with smaller transverse dimensions generally exhibited higher maximum torques.

The evaluation of the performance and utility of the examined appliances underscored the effectiveness of the WQH in facilitating tooth movements. This appliance consistently demonstrated superior characteristics compared to both the WPA and RQH, notably by exerting significantly reduced forces and minimizing torque development, even during an 8 mm expansion distance deemed unsuitable for the other appliances. A distinctive advantage of the WQH moreover lies in its capability to apply root torque, a feature not typically available in traditional quadhelices such as the RQH. Conversely, the evaluation of the WPA highlighted its limitations for extensive tooth movements due to its propensity for generating high force and torque values across all three simulated movements.

While the diverse size range of Wilson^®^ 3D^®^ appliances allows for tailored selection according to individual patient anatomical considerations, careful attention must be given to maintaining an appropriate force-to-torque ratio, particularly for smaller appliance sizes.

The current investigation undeniably has specific methodological shortcomings that require particular discussion. Although the molar locks were precisely positioned on the Frasaco teeth to enable a stress-free insertion of the appliances and reduce initial forces prior to the commencement of the trial, minor deviations in positioning were unavoidable. These deviations could influence the force system during the trial. Additionally, the appliances underwent minimal pre-activation through a bending process to facilitate their insertion, resulting in an increase in the initial forces. Another limitation of this study was certainly the use of only two Frasaco teeth, which eliminated influencing factors such as force distribution on adjacent teeth. A more realistic approach would involve a dental arch model with 14 teeth, allowing for the placement of brackets and arches in the simulation. However, considering the extreme complexity of FE simulations, a deliberate decision was made to concentrate on the force distribution of these two teeth and exclude the variables introduced by neighboring teeth at this stage.

An additional constraint of this study lies in the experimental test methodology. The activation of tooth movement was conducted using the OMSS, which enables the simulation of realistic tooth movements, thus providing valuable information about orthodontic treatment. However, the OMSS cannot fully replicate an intraoral environment and its influencing factors on the experimental force system. Consequently, elements such as soft tissues, musculature, periodontium, and the resulting physiological movement patterns, along with masticatory forces, are not accurately represented within this system. Therefore, the experimentally simulated examination can only partially approximate a clinical situation. Despite these constraints, the clinical relevance of the OMSS remains significant, as it is the only experimental method to date allowing for the recording of both static and dynamic force systems [14].

Taken together, the OMSS facilitated a clinically proximate assessment of the applied force systems, underlining the necessity to acknowledge that this study relies solely on experimentally simulated tooth movements, which inherently restricts the precision of statements regarding force and torque development under intraoral conditions. Consequently, the measured values should not be directly extrapolated to clinical practice without careful consideration but rather used as indicative information. Subsequent clinical studies are imperative to validate or challenge the findings regarding the appliances investigated aiming to implement precise and realistic insights into orthodontic treatment. Nevertheless, the data acquired in this study serve as a foundational framework for these future investigations, setting the stage for more comprehensive and conclusive research approaches in this field. Efforts to optimize appliance dimensions in alignment with therapeutic guidelines are essential to achieving predictable and beneficial orthodontic outcomes while minimizing potential risks to dental health.

## 5. Conclusions

In conclusion, this study highlights the significance of understanding biomechanical principles in orthodontic treatments to attenuate unintentional tooth movements as much as force and torque generation. By systematically comparing orthodontic appliances acting in the transverse plane, the findings provide valuable insights into the force systems of orthodontic appliances and lay the foundation for future clinical studies, aiming to refine and validate the application of these in vitro results.

## Figures and Tables

**Table 1:** The transversal dimensions of the appliances investigated for each of the Wilson^®^ 3D^®^ Quadhelix (WQH), Wilson^®^ 3D^®^ Multi-Action Palatal Appliance (WPA), and Remanium^®^ Quadhelix (RQH).

Appliance	Size/Transversal Dimension
WPA	32 mm 36 mm 40 mm 44 mm 50 mm 56 mm
WQH	25 mm 27 mm 31 mm 34 mm 37 mm 40 mm
RQH	36 mm 38 mm 40 mm

**Table 2:** An overview of the test series conducted for the Wilson^®^ 3D^®^ Quadhelix (WQH), Wilson^®^ 3D^®^ Multi-Action Palatal Appliance (WPA), and Remanium^®^ Quadhelix (RQH).

Test Series	Appliance	Simulated Tooth Movement	Activation Distance
**1.1**	WPA	Expansion	6 mm, 8 mm
**1.2**	WQH	Expansion	8 mm
**1.3**	RQH	Expansion	8 mm
**2.1**	WPA	Rotation	10°
**2.2**	WQH	Rotation	10°
**2.3**	RQH	Rotation	10°
**3.1**	WPA	Root torque	10°
**3.2**	WQH	Root torque	10°

**Table 3:** A compilation of the measured force values of all devices and comparative recommendation of the force values from the literature [15]. Abbreviations used: Wilson^®^ 3D^®^ Quadhelix (WQH), Wilson^®^ 3D^®^ Multi-Action Palatal Appliance (WPA), and Remanium^®^ Quadhelix (RQH).

Tooth Movement	Recommended Force Size for Molars	Measured Force Sizes for Molars		
		WPA	WQH	RQH
Expansion	1.2 N	8.5–>15.0 N	2.7–12.6 N	3.5–5.2 N
Rotation	0.6 N	3.1–6.1 N	0.1–1.7 N	1.4–1.8 N
Root torque	1.0 N	3.9–5.1 N	0.9–2.9 N	

**Table 4:** A comparison of the force results of the simulated expansion for all appliances investigated with regard to significant differences after statistical analysis using Student’s *t*-tests and Bonferroni correction. Abbreviations: Wilson^®^ 3D^®^ Multi-Action Palatal Appliance (WPA), Wilson^®^ 3D^®^ Quadhelix (WQH), and Remanium^®^ Quadhelix (RQH). s: significant (*p* < 0.00048); ns: not significant (*p* > 0.00048).

		WPA						WQH						RQH		
	*Size*	*32*	*36*	*40*	*44*	*50*	*56*	*25*	*27*	*31*	*34*	*37*	*40*	*36*	*38*	*40*
**WPA**	***32***	-	s	s	s	s	s	s	s	s	s	s	s	s	s	s
	***36***	-	-	s	s	s	s	s	s	s	s	s	s	s	s	s
	***40***	-	-	-	s	s	s	s	s	s	s	s	s	s	s	s
	***44***	-	-	-	-	s	s	ns	s	s	s	s	s	s	s	s
	***50***	-	-	-	-	-	ns	s	s	ns	s	s	s	s	s	s
	***56***	-	-	-	-	-	-	s	s	s	s	s	s	s	s	s
**WQH**	***25***	-	-	-	-	-	-	-	ns	s	s	s	s	s	s	s
	***27***	-	-	-	-	-	-	-	-	s	s	s	s	s	s	s
	***31***	-	-	-	-	-	-	-	-	-	s	s	s	s	s	s
	***34***	-	-	-	-	-	-	-	-	-	-	s	s	ns	s	s
	***37***	-	-	-	-	-	-	-	-	-	-	-	s	s	s	s
	***40***	-	-	-	-	-	-	-	-	-	-	-	-	s	s	s
**RQH**	***36***	-	-	-	-	-	-	-	-	-	-	-	-	-	s	s
	***38***	-	-	-	-	-	-	-	-	-	-	-	-	-	-	s
	***40***	-	-	-	-	-	-	-	-	-	-	-	-	-	-	-

**Table 5:** A comparison of the torque results of the simulated expansion for all appliances investigated with regard to significant differences after statistical analysis using Student’s *t*-tests and Bonferroni correction. Abbreviations: Wilson^®^ 3D^®^ Multi-Action Palatal Appliance (WPA), Wilson^®^ 3D^®^ Quadhelix (WQH), and Remanium^®^ Quadhelix (RQH). s: significant (*p* < 0.00048); ns: not significant (*p* > 0.00048).

		WPA						WQH						RQH		
	*Size*	*32*	*36*	*40*	*44*	*50*	*56*	*25*	*27*	*31*	*34*	*37*	*40*	*36*	*38*	*40*
**WPA**	***32***	-	ns	ns	ns	s	s	s	s	s	s	s	s	s	s	s
	***36***	-	-	ns	ns	s	s	s	s	s	s	s	s	s	s	s
	***40***	-	-	-	ns	s	s	s	s	s	s	s	s	s	s	s
	***44***	-	-	-	-	s	s	ns	s	s	s	s	s	s	s	s
	***50***	-	-	-	-	-	ns	ns	ns	s	s	s	s	ns	ns	ns
	***56***	-	-	-	-	-	-	ns	s	s	s	s	s	s	ns	s
**WQH**	***25***	-	-	-	-	-	-	-	ns	s	s	s	s	ns	ns	ns
	***27***	-	-	-	-	-	-	-	-	s	s	s	s	s	ns	ns
	***31***	-	-	-	-	-	-	-	-	-	s	s	ns	s	s	s
	***34***	-	-	-	-	-	-	-	-	-	-	ns	s	ns	s	s
	***37***	-	-	-	-	-	-	-	-	-	-	-	s	s	s	s
	***40***	-	-	-	-	-	-	-	-	-	-	-	-	s	s	s
**RQH**	***36***	-	-	-	-	-	-	-	-	-	-	-	-	-	ns	s
	***38***	-	-	-	-	-	-	-	-	-	-	-	-	-	-	ns
	***40***	-	-	-	-	-	-	-	-	-	-	-	-	-	-	-

## Data Availability

The original contributions presented in the study are included in the article, further inquiries can be directed to the corresponding author/s.
